# Exploring differences in patient participation in simulated emergency cases in co-located and distributed rural emergency teams – an observational study with a randomized cross-over design

**DOI:** 10.1186/s12873-024-01037-3

**Published:** 2024-07-15

**Authors:** Hanna Dubois, Tanja Manser, Henrike Häbel, Maria Härgestam, Johan Creutzfeldt

**Affiliations:** 1 Department of Clinical Science, Intervention and Technology, Karolinska Institutet, K32 Karolinska University Hospital, Stockholm, S-14186 Sweden; 2 Department of Clinical Science and Education, Karolinska Institutet, Södersjukhuset, Stockholm, S-11883 Sweden; 3 FHNW School of Applied Psychology, FHNW University of Applied Sciences and Arts Northwestern Switzerland, Riggenbachstrasse 16, CH-4600 Olten, Switzerland; 4 Department of Learning, Informatics, Management and Ethics, Medical Statistics Unit, Karolinska Institutet, C7, Stockholm, S-171 77 Sweden; 5 Department of Nursing, Umeå University, S-907 87 Umeå, Sweden

**Keywords:** Cottage hospital, Emergency care, Observational study, Patient-centered care, Patient participation, Rural healthcare, Shared decision-making, Teamwork, Telemedicine

## Abstract

**Background:**

In northern rural Sweden, telemedicine is used to improve access to healthcare and to provide patient-centered care. In emergency care during on-call hours, video-conference systems are used to connect the physicians to the rest of the team – creating ‘distributed teams’. Patient participation is a core competency for healthcare professionals. Knowledge about how distributed teamwork affects patient participation is missing.

The aim was to investigate if and how teamwork affecting patient participation, as well as clinicians’ perceptions regarding shared decision-making differ between co-located and distributed emergency teams.

**Methods:**

In an observational study with a randomized cross-over design, healthcare professionals (*n* = 51) participated in authentic teams (*n* = 17) in two scripted simulated emergency scenarios with a standardized patient: one as a co-located team and the other as a distributed team. Team performances were filmed and observed by independent raters using the PIC-ET tool to rate patient participation behavior. The participants individually filled out the Dyadic OPTION questionnaire after the respective scenarios to measure perceptions of shared decision-making. Scores in both instruments were translated to percentage of a maximum score. The observational data between the two settings were compared using linear mixed-effects regression models and the self-reported questionnaire data were compared using one-way ANOVA. Neither the participants nor the observers were blinded to the allocations.

**Results:**

A significant difference in observer rated overall patient participation behavior was found, mean 51.1 (± 11.5) % for the co-located teams vs 44.7 (± 8.6) % for the distributed teams (*p* = 0.02). In the PIC-ET tool category ‘Sharing power’, the scores decreased from 14.4 (± 12.4) % in the co-located teams to 2 (± 4.4) % in the distributed teams (*p* = 0.001). Co-located teams scored in mean 60.5% (± 14.4) when self-assessing shared decision-making, vs 55.8% (± 15.1) in the distributed teams (*p* = 0.03).

**Conclusions:**

Team behavior enabling patient participation was found decreased in distributed teams, especially regarding sharing power with the patient. This finding was also mirrored in the self-assessments of the healthcare professionals. This study highlights the risk of an increased power asymmetry between patients and distributed emergency teams and can serve as a basis for further research, education, and quality improvement.

**Supplementary Information:**

The online version contains supplementary material available at 10.1186/s12873-024-01037-3.

## Background

Residents of rural areas often have long travel distances to care facilities and other health services. Along with technological advancement and digitalization across various healthcare settings, innovative rural care models rely on telemedicine to ‘reduce’ the distances between patients and their caregivers, also often with the ambition to provide patient-centered care [1, 2].

In Västerbotten county, in northern inland Sweden, emergency care has turned towards telemedicine (i.e., information and communication technologies used for healthcare services over distance) for access to medical expertise, regardless of geographical distance [3]. In this rural setting, emergency care is provided in so called ‘cottage hospitals’, i.e., primary healthcare centers, with both out- and in-patient care. Patients in need of in-hospital care can either be admitted to the small cottage hospital ward or transferred to a tertiary hospital. In the cottage hospitals, examples of telemedicine solutions are tele-otoscopes, X-ray images transferred for a radiology’s assessment overseas to benefit from time difference, and stethoscopes with a wireless computer connection for remote assessments. During on-call hours, physicians can be connected to the rest of the team by using a video-conferencing system. This creates so called ‘distributed teams’ in contrast to co-located teams with all team members on site. ECGs and lab results are digitally transferred to the electronic healthcare record and thereby accessed by a remote physician if needed.

In rural areas, telemedicine has previously been associated with high levels of satisfaction in both patients and healthcare professionals, especially regarding improved access to care and reduced travel time [4, 5]. A reported downside of ‘virtual’ patient-clinician meetings is, however, that it is perceived as less personal [6, 7]. In the Swedish cottage hospital context, the aim has not been to replace the physical emergency care by video conference solutions, but rather complement the team of nurses and assistant nurses with the medical expertise missing on site – thus combining ‘the best of both worlds’. The knowledge on provider and patient experiences and satisfaction of this model of emergency care is limited.

In the research field of ‘non-technical skills’, teamwork, communication, and patient safety are commonly studied, especially within surgical and emergency settings [8, 9]. Patient participation, however, is a largely overlooked domain in this research field. Patient participation is recognized worldwide as an ethical imperative, partly to strengthen patient safety [10] and as a part of the core competency, patient-centered care, which applies to all healthcare professionals [11]. The patient can contribute with valuable information about their health and life situation, including symptoms, medical history and heredity, but also communicate their expectations of care. Patient participation in general has been described as a reciprocal and caring patient-professional relationship, information exchange and shared decision-making [12–14]. In emergency care, also aspects such as comfort, respect, emotional support and trust have been highlighted as important for patient participation [15]. Knowledge about patient participation in emergency care settings is, however, based on co-located care teams. Patient participation in distributed emergency teams is an unexplored field yet. For domains of healthcare introducing video conference solutions, it is important to understand the implications of such novel teamwork settings for multiple dimensions of care, including patient participation.

In a previous interview study, rural emergency care professionals reported that they experienced the team dynamics in the distributed team setting as altering the patients’ possibilities for participation [7]. Healthcare professionals’ attitudes, behavior and the well-functioning multi-professional care team have been reported to be crucial conditions for patient participation [16, 17]. As patient participation is mostly described in one-to-one patient-clinician encounters, also exploring team behavior in an emergency context is relevant. Knowledge on distributed team behavior affecting patient participation, and how it may differ from co-located teams, is largely missing and thus needs to be investigated.

## Methods

### Aim

This study aimed to investigate *if* and *how* the team setting (i.e., distributed using tele-medicine vs co-located) impacts on clinicians’ behaviors and perceptions related to patient participation in rural emergency care.

## Research questions

Q1. What differences in team behavior related to patient participation between co-located teams and distributed teams can be observed?

Q2. Do healthcare professionals perceive shared decision-making, as a key component of patient participation, differently in a distributed team setting than in a co-located team setting?

### Study design

To compare differences in healthcare providers’ behaviors and perceptions regarding (markers of) patient participation, a randomized cross-over design was used (Supplement 1). The two conditions were co-located and distributed team settings.

**Fig. 1:**
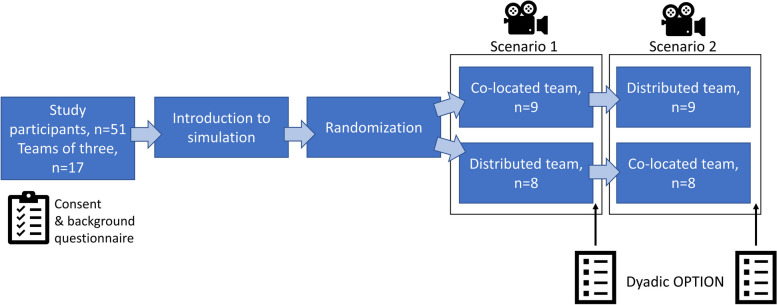
Flow chart of data collection

Emergency care simulations with a standardized patient (actor) were used for data collection. Rural healthcare teams acted in two simulations: as a co-located team (i.e., all team members in the same room with the patient) and as a distributed team (i.e., physician participating by video-conferencing system). Teams were randomized to either start in the co-located or distributed condition to even out potential learning effects between the scenarios (Fig. 1). The simulations were video-recorded. Several of these teams also participated in an interview study in the same research project [7]. Data collection was carried out between September 2019 and November 2021.

### Sample and setting

All cottage hospitals (*n* = 7) in the rural inland areas of the county of Västerbotten in northern Sweden were included in the study. The cottage hospitals include a primary healthcare center at daytime, a 24/7 emergency room, a small in-patient ward, radiology equipment and a small laboratory. During on-call hours, a nurse and nurse assistant staff the emergency room. The physician on-call is responsible for several cottage hospitals and can be consulted over telephone or a video-conferencing system.

The video-conferencing system installed in the emergency rooms at the time of the study was Cisco TelePresence SX20 system, i.e., a camera catching and angle of the entire room (default choice), a hand-maneuvered camera which could be used for close-up images (optional), and a large screen fixed on a wall with an integrated audio-system. A remote user of this system would thus connect to the emergency room from a laptop computer or another conference system. A close-up face view of the remote user would be displayed on the emergency room screen. The video conference system included speakers, which made communication overt for everyone in the room.

Healthcare professionals working at the cottage hospitals were invited to participate in the study. Prior to the simulations, all participants received written and oral information about the study, filled out a background questionnaire and consented to participation in the study.

#### Participants

Healthcare teams (*n* = 17) from all seven cottage hospitals participated in the study. Each team consisted of three healthcare professionals: a physician, a nurse and either a nurse assistant or a second nurse. Participant characteristics are presented in Table 1.

**Table 1:** Participant characteristics

	n (%)	Age, mean/median (range)	Gender, n (%)	Professional experience in healthcare, mean/median (range)	Previous experience of tele-medicine, n (%)	Specialist degree, n (%)
All	51 (100)	42.6/42 years (20–69)	Female 42 (82.4) Male 9 (17.6)	17.7/16 years (8 months-44 years)	14 (27.5)	17 (33.3)
Nurse assistants	13 (25.5)	39.1/37 years (20–64)	Female 12 Male 1	17.7/19.75 years (8 months-39 years)^a^	2	0
Nurses	21 (41.2)	41.5 /38 years (23–62)	Female 19 Male 2	17.1/16 years (10 months-44 years)	7	5
Physicians	17 (33.3)	46.8/43 years (36–69) (missing value, *n* = 1)	Female 11 Male 6	18.4/17 years (5–36 years)^a^	5	12

^a^one missing value

#### Simulated emergency scenarios

Simulations were carried out *in-situ* (i.e., at the participants’ respective workplaces). Two emergency scenarios had been developed to represent realistic emergency cases in the rural cottage hospitals, including psychosocial aspects. A multi-professional group of clinicians including physicians and nurses with expertise in rural health care, family medicine, emergency care, pre-hospital care, internal medicine, cardiology and anesthesiology reviewed the scenarios and confirmed their credibility and comparability in medical urgency and demand of resources. Case 1 illustrated a patient with urosepsis and case 2 a patient with an ongoing myocardial infarction. More detailed descriptions of the cases are provided in Supplement 2.

Case 1 was used for the co-located team setting and Case 2 for the distributed teams. In both cases, the expected medical decisions were to start treating the patient (antibiotics and fluids in Case 1 and thrombolysis in Case 2) and then transfer the patient to the tertiary hospital, either by road transport (ambulance) or by helicopter (air ambulance). A facilitator provided information about the patient when requested by the participants (e.g., body temperature, plasma glucose and other point of care lab results). The facilitator also provided values displayed on a monitor for vital parameters (blood pressure, heart rate, peripheral oxygen saturation). ECG and medical records were provided on paper and not on the electronic medical healthcare record as used in the cottage hospitals. The participants could administer intravenous drugs or push fluids by using an iv-line on the standardized patient’s arm, which was connected to a hidden reservoir. The standardized patient followed a script enabling the participants to engage in a conversation with her. The patient wore a skin-colored t-shirt underneath her clothes which the participants were instructed to consider as a bare chest in case of need to undress her. The scenarios were discontinued when the teams had initiated treatment and planned for the medical care of the patient.

### Measures of patient participation

#### Behavioral rating

Video-observations were performed by two independent observers using the PIC-ET tool [18], a 22-item observation tool for assessment on patient involvement and collaboration (PIC) behavior in emergency care teams from a third person perspective. This instrument was previously developed and tested by our research group. Inter-rater reliability was assessed as ‘fair’ (Kappa 0.52). Items in the PIC-ET tool are grouped in five categories: ‘Relationship’, ‘Sharing power’, ‘Information exchange’, ‘Safe and caring environment’ and ‘Social circumstances’. Behaviors described in each item are scored on verbally anchored four-point scales (for five items only two options) on patient involvement and collaboration, ranging from ‘no PIC’ to ‘high PIC’.

#### Self-assessment

No validated self-assessment instrument fully covers all dimensions of patient participation or its neighboring concepts [19]. The Dyadic OPTION [20] was, thus, chosen to measure the participants’ perspectives on shared decision-making, a core component of patient participation, frequently reported as relevant in emergency care [21, 22]. The Dyadic OPTION, a validated 12-point questionnaire [20], is based on the OPTION instrument [23], which has been extensively validated and found to have both strong internal consistency, as well as intra- and interrater reliability [24].The Dyadic OPTION was found to correlate with the original instrument (coeff. 0.58) [25] but shifted focus to the unique perspectives of the parties in a care encounter (patient and clinician). The Dyadic OPTION has been found to be the most promising and reliable tool in measuring elements of patient participation [24]. Although this instrument can be used to measure both patients’ and clinicians’ perceptions, due to the use of a standardized patient in our study, only the professionals’ perspective was measured by asking them to individually fill out the questionnaire at the end of each scenario.

### Statistical analysis

Participant characteristics were described as number and percentage for categorical variables and as means, medians and range for numerical variables. For the outcome variables, total and category-specific scores were translated to percentage of maximum score and are presented as mean and standard deviation (SD). The percentage of maximum score was calculated by translating the lowest possible score to 0% and the highest possible score to 100%. All intermediate possible scores were distributed evenly spaced between 0 and 100%. Total scores and category-specific scores were obtained by dividing the observed sum of all item scores with the largest possible sum. If there was any missing item score, no total score was calculated. A boxplot was used to describe and present the distribution of Dyadic OPTION, total score.

Differences in PIC-ET tool mean total and category-specific scores between co-located and distributed teams were tested using linear mixed-effects regression models allowing for a random intercept per team. The models included setting (reference distributed teams), rater and an interaction term between setting and rater. Visual inspection of histograms and residual plots were used to assess the distribution of the outcome variables and model residuals. Due to an observed skewed distribution, a generalized linear mixed-effects model with a gamma family was used for the category ‘Sharing power’. The robust sandwich estimator was used to estimate standard errors. In a sensitivity analysis, weighted scores were considered with weights according to expert ratings described previously [18]. In another sensitivity analysis, missing item scores were imputed with the average category-specific score.

Differences in perceptions of shared decision-making between co-located and distributed teams were tested using a paired t-test. Differences in mean Dyadic OPTION score between any of the professions were tested using one-way ANOVA in the co-located and distributed teams, respectively. Pairwise comparisons for each professional group between team setting were made in a post-hoc analysis using the Sidak multiplicity correction after fitting a linear mixed model including the variables team setting and professional group as well as an interaction term between the two.

Two-sided *p*-values were reported and a *p*-value of 0.05 was considered statistically significant. All analyses were conducted using STATA version 16.1 (Stata Corp, College Station, Texas, USA).

## Results

The lengths (min:sec) of co-located team scenarios were 09:49–19:31 (range), 14:09 (mean), 13:56 (median), and 12:02–22:00 (range), 15:56 (mean), 16:08 (median) for the distributed team scenarios.

### Patient participation behavior by the care team

We found a significant difference in observer rated overall patient participation behavior, 51.1 (± 11.5) % for the co-located teams vs 44.7 (± 8.6) % for the distributed teams (*p* = 0.02) (Table 2).

**Table 2:** PIC-ET tool mean scores (SD), translated to percentage of maximum score on all items and the five included categories

	**Observations all items, n**	**All items**	**Relationship**	**Sharing power**	**Information exchange**	**Safe and caring environment**	**Social circumstances**
Co-located	23	51.1 (11.5)	60.5 (17.8)	14.4 (12.4)	49.0 (15.7)	56.3 (22.0)	62.0 (22.3)
Distributed	19	44.7 (8.6)	56.2 (12.6)	2.0 (4.4)	44.5 (16.6)	51.6 (19.3)	46.8 (24.4)
*p*-value		0.02	ns	0.001	ns	ns	ns

Results from the conducted sensitivity analyses were in line with the findings from the main analyses (results not shown).

### Perceptions of shared decision-making

**Fig. 2:**
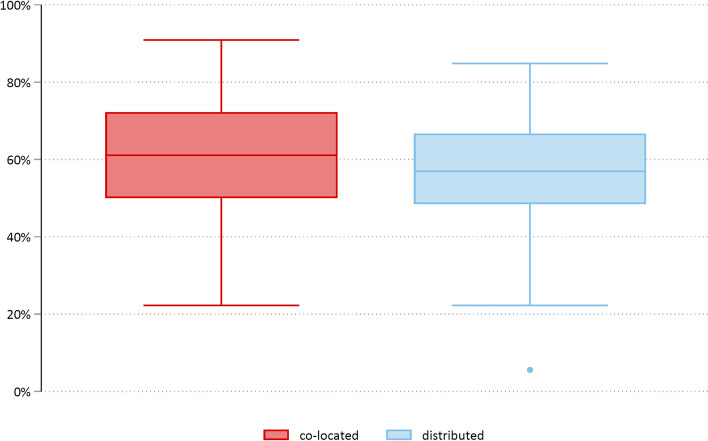
Box plot, Dyadic OPTION, total score distribution for co-located and distributed teams displaying minimum, 25th percentile, median, 75th percentile, maximum; dots indicate observations more than 1.5 times the inter quartile range away from the 25th or 75th percentile

Dyadic OPTION, total scores were distributed symmetrically for co-located and distributed teams with apparent minor differences in spread (slightly larger for co-located teams) and center (slightly smaller for distributed teams) (Fig. 2).

In mean, co-located teams scored 60.5% (± 14.4) when self-assessing shared decision-making, vs 55.8% (± 15.1) in the distributed teams (*p* = 0.03). The scores did not differ significantly between the professions in the co-located teams, however, there was a statistically significant difference in the distributed teams between the professions, i.e., the physicians rated shared decision-making lower than nurses and nurse assistants (Table 3). One item (# 9, ‘The patient had opportunities to ask questions’) was rated lower in the distributed teams (regression coeff. -2.04, non-multiplicity adjusted *p*-value 0.012).

**Table 3:** Dyadic OPTION, mean (SD) percentage scores

Team composition	Physicians, *n* = 17	Nurses, *n* = 21	Nurse assistants, *n* = 13	*p*-value*
Co-located	53.9 (SD 13.1)	62.6 (14.5)	65.9 (13.7)	0.05
Distributed	47.8 (SD 16.8)	58.7 (12.0)	61.7 (13.5)	0.02
*p*-value**	ns	ns	ns	

^*^One-way ANOVA

^**^Post-hoc pairwise comparisons with Sidak correction after fitting linear mixed model

## Discussion

In this study, in a rural emergency setting, we found higher scores for team behavior related to patient participation for co-located teams than for distributed teams. In particular, ratings of observed behaviors associated with shifting power from the professionals to the patient were significantly lower in the distributed team setting. This finding was also mirrored in the questionnaires measuring the clinicians’ perceptions of shared decision-making. The results imply that the distributed emergency team setting may reduce the patients’ position of power and thus, inhibit patient participation, especially regarding shared decision-making. To our knowledge, this is the first study comparing patient participation between co-located and distributed rural emergency care teams.

In our previous work, when interviewing healthcare professionals working in distributed emergency teams, team dynamics were described to be affected when using telemedicine solutions, i.e., the physician and the patient were perceived to be pushed further apart, leaving the patient in an exposed position. In particular, decisions about admission or transfer to the hospital were described by physicians to be difficult to make together with the patient in a distributed setting [7]. In the current study, shared decision-making was rated significantly lower in the distributed setting by physicians than by nurses and nurse assistants. This is in line with the previous interview findings [7] and may imply that the physician–patient relationship is especially vulnerable when the physician is physically separated from the rest of the team.

Delivering patient-centered care, which includes patient participation and shared decision-making, is a highly valued competency for all healthcare professionals [10, 11]. While shared decision-making in ‘virtual’ appointments has been shown to be comparable to in-person visits in other areas of healthcare [1, 26], literature on shared decision-making in an emergency care team context involving telemedicine is missing. In this study, we observed low levels of ‘sharing power’, including shared decision-making, in both settings. This is consistent with previous research on shared decision-making in emergency settings. Schoenfeld et al. found that less than half of emergency patients who were physically and cognitively stable reported that they were involved in decision-making [27]. Given the already low levels of shared decision-making in emergency care settings in general, as well as in the co-located setting in this study, the substantial decrease in the distributed setting regarding sharing power is worrying and should be further investigated. Exploring communication patterns within distributed emergency teams in relation to patients could be useful to identify means to strengthen the patient-physician relationship, and thus, at least partly, even out the power asymmetry between patients and emergency teams. It would also be of interest to investigate how teams relying on telemedicine respond to additional technology (e.g., ECG telemetry or other non-communicative data-transfers) in terms of patient involvement. Does additional technology free up resources to concentrate on communication with the patient or is it a disturbance for the teams?

Although telemedicine in rural emergency care has its advantages, e.g., increased access to medical expertise in remote areas, our study indicates that patient participation may be at risk when introducing distributed teamwork for healthcare professionals trained to work in co-located team settings. From a healthcare professional point-of-view, the patient-physician relationship suffers from the remoteness, however, this needs to be assessed also in teams with more substantial experience from distributed emergency team work as well as from a patient perspective.

Disparities between rural and urban emergency and trauma care have previously been reported, i.e., rural populations do not have the same access to emergency care as their urban counterparts, due to distances and shortage of staff [28]. Rural healthcare professionals have been described to carry a clinical responsibility of complex nature and thus, require education and training adapted to their unique work environment [3, 29]. Our study sheds light on aspects relevant to patient participation when introducing distance solutions in emergency care in a rural area and highlights the risk of weakening the patient position in this setting. This new knowledge is important when tailoring education for emergency care teams relying on telemedicine and motivates the development and implementation of technology supporting differing needs of communication in emergency care. When evaluating new models of healthcare delivery, areas of improvement can be detected. This study has pointed out risks of distributed teamwork in emergency care and may serve as a basis for future research, education and quality improvement.

### Strengths and limitations

A strength in our randomized cross-over study design was the standardized and scripted simulations. The scenarios were thoroughly developed for realism and comparability. The participants were authentic teams, working together on a daily basis and we found them highly motivated to participate in the study (no loss of subject). A trained patient during the simulations instead of a patient simulator contributed to credibility, and the study participants could naturally interact with the patient. Simulations enable observations of emergency teams in action, without compromising patient integrity. Also, collecting real-life data of this kind in this geographical area would be very difficult, as there are low volumes of emergency patients and their occurrence is unpredictable. Systematic variation of setting with cases of comparable urgency would not be possible to collect in real-life clinic.

This study is not without limitations. The data collection was challenging and lengthy, due to the limited access to available healthcare teams in this rural area and to the covid-19 pandemic. Some data was also lost when the raters had found an item non-applicable for the situation observed, which decreased the number of cases available for analysis. Although significant difference overall between co-located and distributed teams, on a category-level the only significant difference in observed behavior was in ‘Sharing power’. However, we cannot rule out that there are differences in other categories in the PIC-ET tool, which could possibly have been detected in a larger study sample, ideally with more diverse medical cases. Also, the PIC-ET tool is a newly developed instrument and its reliability and validity remain yet to be tested more fully. However, findings from the Dyadic-OPTION and interviews with rural healthcare professionals [7] support the observations made with the PIC-ET tool: the distributed team setting attenuates shared decision-making, a key component of patient participation. A limitation to the generalizability of these results is that neither the participants nor the observers could be blinded to the allocations.

## Conclusions

By comparing co-located and distributed emergency teams in a rural setting, team behavior enabling patient participation was found decreased in distributed teams, especially regarding sharing power with the patient. This finding was also mirrored in the self-assessed questionnaires measuring the healthcare professionals’ perceptions on shared decision-making. This new knowledge can be used when further examining interactions between a distributed team and a patient, with the ultimate goal to tailor education for distributed teams and when designing interventions for quality improvements in healthcare.

### Supplementary Information

Supplementary Material 1.

Supplementary Material 2.

## Data Availability

The datasets used and analysed during the current study are available from the corresponding author on reasonable request.
