# Supplementary figures and images for: Exploring differences in patient participation in simulated emergency cases in co-located and distributed rural emergency teams – an observational study with a randomized cross-over design
Source: BMC Emerg Med. 2024 Jul 15;24:118. doi: 10.1186/s12873-024-01037-3 (PMC11247836; doi:10.1186/s12873-024-01037-3)

# Flow chart of the randomized cross-over design of the study

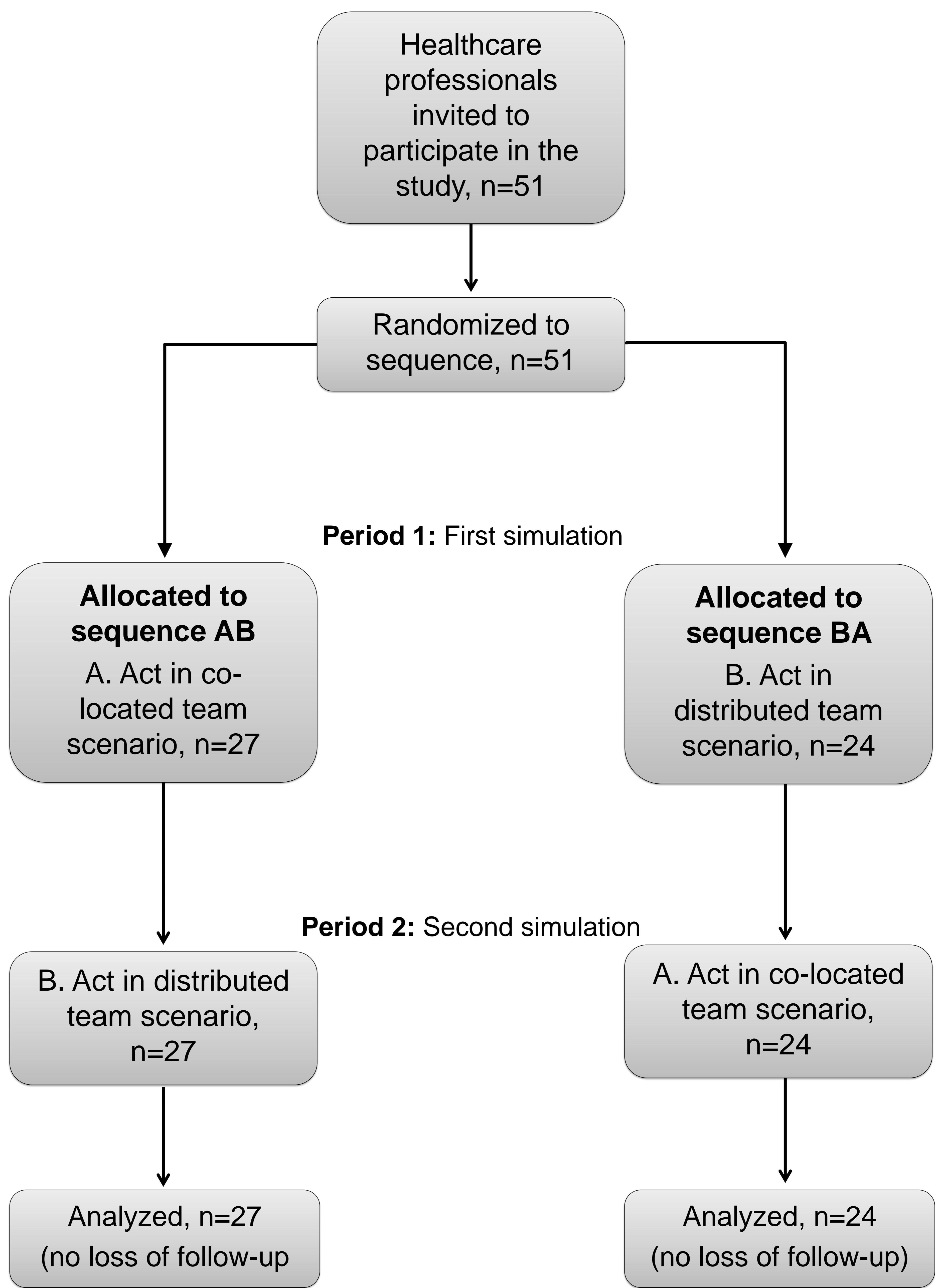

Supplement: Supplementary file 1 — Supplementary Material 1. [file 12873_2024_1037_MOESM1_ESM.pdf]
