# Supplementary material for: Exploring differences in patient participation in simulated emergency cases in co-located and distributed rural emergency teams – an observational study with a randomized cross-over design
Source: BMC Emerg Med. 2024 Jul 15;24:118. doi: 10.1186/s12873-024-01037-3 (PMC11247836; doi:10.1186/s12873-024-01037-3)
Supplement: Supplementary file 2 — Supplementary Material 2. [file 12873_2024_1037_MOESM2_ESM.pdf]

Case 1 – urosepsis

Female, age 71

Cause of contact: Low abdominal pain since yesterday, burning sensation when urinating. Vomited once, no apparent bleeding. Neighbour drove her to the emergency room.
Medical history: Long-term back pain, recurring urinary tract infections.
Current medication: Paracetamol 1000 mg (when needed)
Social situation: Takes care of wheelchair bound husband. No domestic service. Husband is home alone with their dog. Adult daughter living in Umeå.

**First assessment**	
**A**	free airway
**B**	respiratory rate 20/min, peripheral oxygen saturation 94 %
**C**	blood pressure 90/60 mmHg, pulse 100/min
**D**	awake, uneasy
**E**	fever, body temperature 39.0 degrees Celsius, abdomen is soft upon palpation
**Second assessment (deteriorating)**	
**A**	free airway
**B**	respiratory rate 24/min, peripheral oxygen saturation 94 % without administered oxygen (97 % with administered oxygen)
**C**	blood pressure 80/60 mmHg, pulse 125/min
**D**	passed out, but wakes up during examination
**E**	fever, body temperature 39.0 degrees Celsius, pale, no cyanosis.

Example from script for standardized patient in Case 1

Prompt from patient	Expected team behavior	Reaction/response from patient
‘My belly is hurting, why is it hurting?’	Asks patient to describe pain/ uses numerical rating scale for pain assessment/offers analgesics	Describes pain as intermittent in the lower abdomen, assesses pain to be 7-8 on a 10-point numerical rating scale.
‘I would rather not use antibiotics, they upset my bowel’	Motivates/Explains	Accepts treatment
‘I need to go home to take care of my husband’	Takes social circumstances into account/Proposes to call someone to help him.	Accepts decision of transfer to hospital.

Case 2 – ST depression myocardial infarction

Female, age 70

Cause of contact: Light headedness, chest discomfort and nausea during walk with the dog. Neighbour drove her to the emergency room.
Medical history: Depression and anxiety
Current medication: Citalopram 20 mg x 1/day, oxazepam 5 mg (when needed)
Social situation: Widow, history of sick leave from work due to depression, living on her own with a dog. Visits her old mother daily who depends on her help.

**First assessment**	
**A**	free airway
**B**	respiratory rate 24/min, peripheral oxygen saturation 96 %, shortness of breath
**C**	blood pressure 160/80 mmHg, pulse 111/min, ECG shows ST depression
**D**	anxious
**E**	Normal body temperature, 36.8 degrees Celsius, pale, cold sweat/moist skin, abdomen is soft upon palpation
**Second assessment (deteriorating)**	
**A**	free airway
**B**	respiratory rate 26/min, peripheral oxygen saturation 93 % without administered oxygen (97 % with administered oxygen)
**C**	blood pressure 160/80 mmHg, pulse 120/min, ECG shows ST depression, describes severe chest pain
**D**	anxious, not wanting to talk
**E**	pale, no cyanosis, cold sweat/moist skin

Example from script for standardized patient in Case 2

Prompt from patient	Expected team behavior	Reaction/response from patient
'What is going on?'	Somebody explains	Wants to hold someone's hand
Describes chest pain as severe, 8-9 on a 10-point numerical scale	Starts pharmacological therapy	Pain decreases to 7/10
Worried about the old mother. 'My mother is all by herself'	Takes social circumstances into account/Proposes that someone can contact/visit the mother	Calms down
